# Insight into interplay between PANoptosis and autophagy: novel therapeutics in ischemic stroke

**DOI:** 10.3389/fnmol.2024.1482015

**Published:** 2025-01-08

**Authors:** He-Yan Tian, Yun-Xing Lei, Jing-Tao Zhou, Long-Jun Liu, Tong Yang, Yue Zhou, Jin-Wen Ge, Chen Xu, Zhi-Gang Mei

**Affiliations:** ^1^School of Medical Technology and Nursing, Shenzhen Polytechnic University, Shenzhen, China; ^2^Key Laboratory of Hunan Province for Integrated Traditional Chinese and Western Medicine on Prevention and Treatment of Cardio-Cerebral Diseases, Hunan University of Chinese Medicine, Changsha, China; ^3^Hunan Academy of Traditional Chinese Medicine, Changsha, China

**Keywords:** ischemic stroke, PANoptosis, autophagy, pyroptosis, apoptosis, necroptosis, therapeutics

## Abstract

PANoptosis is a novelly defined mode of programmed cell death that involves the activation of multiple cellular death pathways, including pyroptosis, apoptosis, and necroptosis, triggering robust inflammatory reactions. Autophagy is a crucial cellular process that maintains cellular homeostasis and protects cells from various stresses. PANoptosis and autophagy, both vital players in the intricate pathological progression of ischemic stroke (IS), a brain ailment governed by intricate cell death cascades, have garnered attention in recent years for their potential interplay. While mounting evidence hints at a crosstalk between these two processes in IS, the underlying mechanisms remain elusive. Therefore, this review delves into and dissects the intricate mechanisms that underpin the intersection of PANoptosis and autophagy in this devastating condition. In conclusion, the crosstalk between PANoptosis and autophagy in IS presents a promising target for the development of novel stroke therapies. Understanding the interplay between these two pathways offers a much-needed insight into the underlying mechanisms of IS and opens the possibility for new therapeutic strategies.

## 1 Introduction

Stroke, a leading culprit of mortality and disability in China, causes 5.5 million deaths and 44 million physical disabilities around the world per year (Collaborators, 2019), which is a disease of immense public health importance with serious economic and social consequences (Barthels and Das, 2020; Zhou M. et al., 2019). IS comprises 70–80% of all stroke incidents globally, characterized by disrupted blood flow to the brain tissues. This interruption triggers a complex cascade of pathophysiological events that ultimately leads to various forms of cell death, notably necroptosis, apoptosis, and pyroptosis, among others (Davidson et al., 2020). PANoptosis is a newly described form of programmed cell death that involves the activation of multiple cellular death pathways, including pyroptosis, apoptosis and necroptosis, resulting in a potent inflammatory response. Autophagy is a crucial cellular process that maintains cellular homeostasis and protects cells from various stresses. Recent studies have highlighted the dual role of PANoptosis and autophagy in both initiating and exacerbating brain tissue damage in the aftermath of cerebral ischemia and cerebral ischemia/reperfusion injury (CIRI), emphasizing their intricate involvement in disease progression (Chen et al., 2019; Degterev et al., 2005; Zhang et al., 2020). Tissue-type plasminogen activator (tPA) stands as the sole thrombolytic agent currently approved for clinical application in the management of IS. However, owing to the narrow therapeutic window (<4.5 h) and hemorrhagic transformation, its usage in the treatment of IS has been limited (Alawieh et al., 2018; Henderson et al., 2018). Given the aforementioned reasons, research has delved into interventions that specifically target distinct forms of programmed cell death, aiming to offer innovative therapeutic avenues for IS (Chen et al., 2018; Liu D. et al., 2020). Nonetheless, a growing body of evidence suggests that a substantial interplay exists between PANoptosis and autophagy in the aftermath of IS (Gonzalez-Rodriguez and Fernandez-Lopez, 2023; Wang J. F. et al., 2018; Xu et al., 2017; Yang et al., 2022). This review aims to consolidate the understanding of the intricate mechanisms underlying the crosstalk between PANoptosis and autophagy in IS. Uncovering these mechanisms could potentially unveil novel therapeutic targets, thereby advancing the treatment landscape for IS (Table 1).

**Table 1:** Schematic overview of PANoptosis and autophagy in IS.

	Autophagy	Apoptosis	Pyroptosis	Necroptosis
Morphological characteristics	Intracellular vacuole	Reduced cell size or cellular shrinkage	Cell swelling	Round and swollen cells
Biochemical halmarkers	Autophagosome formation and subsequent degradation process	Initiation of caspase-3/7 activity and subsequent cleavage of its target substrates	Inflammatory caspases trigger GSDMD cleavage, releasing IL-1β and IL-18 cytokines	RIPK1, RIPK3 MLKL activation
Vital regulators	Atg8-I, Atg8-II, Atg9, Atg12, ULK1, mTOR, AMPK, Beclin-1	Bcl-2, Bax, Bid, Caspase cIAP1, HSP70, TNFR1	GSDMD, Caspase, NLRP3, NLRC4, IL-1β and IL-18	RIP1, RIP3, MLKL, Fas/TNFR, p53
Inducers and inhibitors	Inducers: Rapamycin (Tanemura et al., 2012), Chinese herbal medicines including *Ginkgo biloba* extract (Zang et al., 2023) Inhibitors: 3-MA (Seglen and Gordon, 1982). Bafilomycin-A1 (Ryan et al., 2018), Punicalagin (Cao et al., 2019)	Inducers: circCELF1 (Li L. et al., 2023; Li and Teng, 2023) Inhibitors: Z-VAD-FMK (Costa Pereira et al., 2018) BAG3 overexpression (Liu M. et al., 2023), Chinese herbal medicines including Shuan-Tong-Ling (Mei et al., 2017)	Inducers: Ginsenoside Rg1 (Long et al., 2024) Inhibitors: Prussian Blue Nanozyme (Ma et al., 2022), Chinese herbal medicines including Dendrobium alkaloids (Liu D. et al., 2020), Artemisinin (Wang et al., 2024b)	Inducers: alkylating agents (Allocca et al., 2019). Inhibitors: Nec-1 (Zhang et al., 2016), dabrafenib (Cruz et al., 2018), infliximab(Chen et al., 2019), Chinese herbal medicines including curcumin (Wang Y. P. et al., 2017)

## 2 PANoptosis

### 2.1 Exploring the crossroads of pyroptosis, apoptosis, and necroptosis: interactions and regulation

Regulated cell death holds a pivotal role in safeguarding the host. PANoptosis, a newly recognized modality of programmed cell death, encompasses the orchestration of diverse cell death pathways, such as pyroptosis, apoptosis, and necroptosis. Research underscores the existence of an intricate, dynamic molecular interplay among these processes, indicating that their individual cell death mechanisms are not mutually exclusive. Extensive literature has already discussed the distinctions between pyroptosis, apoptosis, and necroptosis. In the following discussion, our primary focus lies in examining the interplay and regulatory mechanisms that govern the relationships between pyroptosis, apoptosis, and necroptosis.

Both pyroptosis and apoptosis involve the activation of Caspase family members, hinting at a shared evolutionary ancestry. Caspases can be categorized into inflammatory caspases (including caspase-1, 4, 5, and 11) and apoptosis-specific caspases (encompassing caspase-3, 6, 7, 8, 9, and 10), further underscoring their distinct yet interconnected roles (Husain, 2022; Kesavardhana et al., 2020a). Nevertheless, Research has revealed that caspase-3 and caspase-8 play pivotal roles in mediating pyroptosis, demonstrating their expanded functional spectrum beyond the realm of apoptosis (Sarhan et al., 2018; Wang C. P. et al., 2017). Caspase-8 is recognized as a versatile molecular switch that governs cell fate, capable of orchestrating apoptosis, pyroptosis, or necroptosis, underscoring its pivotal role in regulating diverse cell death pathways (Fritsch et al., 2019; Jiang et al., 2021).

Gasdermin D (GSDMD) plays a pivotal role in the activation of pyroptosis by cleavage and activation of the protein (Shi et al., 2015). When cells encounter danger signals such as bacterial lipopolysaccharides (LPS) or extracellular ATP, they activate pattern recognition receptors (PRRs) that trigger the formation of inflammasomes. The inflammasomes then activate caspase-1, which cleaves GSDMD at a specific site to release an N-terminal fragment that contains an oligomerization domain. This domain assembles into pore-like structures made up of several GSDMD subunits, causing the cells to lyse and release pro-inflammatory cytokines (Ding et al., 2016).

RIP3 protein (Receptor Interacting Protein Kinase 3) plays a key role in the regulation of necroptosis, which is activated in response to several stimuli, including the binding of certain ligands to TNFR1 (tumor necrosis factor receptor-1), a cell surface receptor involved in inflammation and immunity (Humphries et al., 2015). Upon activation, RIP3 phosphorylates and activates another protein called MLKL (Mixed Lineage Kinase domain-like), which mediates the downstream signaling events that lead to necroptosis.

MLKL, a crucial player in necroptosis, has also been implicated in activating the NLRP3 inflammasome, highlighting its multifaceted role in the immune response and cell death machinery (Frank and Vince, 2019). The study has showed that MLKL can directly activate the NLRP3 inflammasome in response to certain stimuli, leading to the release of mature IL-1β and IL-18 cytokines and the induction of pyroptosis (Sanchez-Fernandez et al., 2019). MLKL can also indirectly activate the NLRP3 inflammasome by promoting the release of mitochondrial DNA (mtDNA) from damaged mitochondria. This mtDNA release triggers the assembly of the NLRP3 inflammasome and the subsequent induction of pyroptosis (Han et al., 2019).

### 2.2 Central molecules in PANoptosis and PANoptosome assembly

#### 2.2.1 Z-DNA-binding protein 1

ZBP1 is an essential cytoplasmic protein involved in a range of cellular functions, including immune responses, cell death, and RNA metabolism. When activated, ZBP1 triggers a complex sequence of events that can lead to various forms of programmed cell death, namely necroptosis, apoptosis, and pyroptosis, collectively known as PANoptosis. This activation process engages key signaling molecules, such as receptor-interacting protein kinase 3 (RIP3), caspase-8, and the NLRP3 inflammasome (Kesavardhana et al., 2020b; Zheng et al., 2020; Zheng and Kanneganti, 2020). ZBP1 is recognized as a vital component of the PANoptosome, a multiprotein complex that regulates these cell death pathways. The PANoptosome is composed of various receptors, including ZBP1 and inflammasome components, along with adapters like ASC (apoptosis-associated speck-like protein containing a CARD) and FADD (Fas-associated protein with death domain), as well as catalytic effectors such as caspase-1, RIP3, RIP1, and caspase-8. This assembly acts as a central hub for integrating signals that determine cell fate under stress conditions. A key aspect of ZBP1 is its Zα2 domain, which is crucial for activating NLRP3 and initiating PANoptosis. This underscores ZBP1’s pivotal role in the regulatory framework governing these cell death processes. When ZBP1 or its Zα2 domain is lost or impaired, significant consequences arise: activation of NLRP3 is markedly reduced, leading to decreased pyroptosis, which is characterized by the release of pro-inflammatory cytokines. Additionally, there is a reduction in the cleavage of executioner caspases, such as caspase-3, caspase-7, and caspase-8, which are vital for the apoptotic process. Moreover, the phosphorylation of MLKL (mixed lineage kinase domain-like protein), an important event in necroptosis, is also diminished. These findings highlight the critical role of ZBP1 and its Zα2 domain in the regulation of PANoptosis. By activating essential pathways involved in cellular stress responses, ZBP1 contributes to maintaining cellular homeostasis and plays a significant role in the immune response to pathogens and cellular injury. A deeper understanding of ZBP1’s multifaceted functions and its interactions within the PANoptosome could offer valuable insights for developing therapeutic strategies targeting diseases associated with dysregulated cell death.

#### 2.2.2 RIP1

RIP1 Regulation in PANoptosis: Essential for Cell Death and Inflammatory Responses (Lalaoui et al., 2020; Tao et al., 2020). In the context of PANoptosis, RIP1 undergoes intricate regulation, exhibiting dualistic roles that are highly context-dependent. Its function alternates between fostering cellular survival and inducing cell death, contingent upon the specific cellular milieu and the presence of regulatory molecules. The loss of RIP1 can trigger RIP3-mediated PANoptosis via caspase-8 and FADD, ultimately resulting in cell death (Dillon et al., 2014). The functionality of RIP1 is further influenced by phosphorylation at multiple sites, which triggers distinct cellular outcomes. Under specific conditions, the phosphorylation of RIP1 promotes the formation of the necrosome, a necessary step for initiating necroptosis. This modification serves as a signal for recruiting additional proteins involved in necroptosis, leading to a cascade of events that ultimately result in cell death. Conversely, dephosphorylated RIP1 enhances its interaction with caspases, guiding the cell toward apoptosis. This dual role highlights the complexity of RIP1’s involvement in determining cell fate. Additionally, various regulatory proteins are essential for modulating RIP1’s activity. Cellular inhibitors of apoptosis proteins (cIAPs) ubiquitinate RIP1, enhancing its pro-survival effects by preventing degradation and promoting survival signaling pathways. In contrast, the deubiquitinating enzyme CYLD removes ubiquitin chains from RIP1, thereby activating mechanisms that lead to cell death. This dynamic interaction between cIAPs and CYLD is crucial for balancing cell survival and death, underscoring the finely tuned regulatory networks that control RIP1’s function. In essence, RIP1’s intricate regulation in PANoptosis underscores the complexity of this programmed cell death and emphasizes the imperative for deeper research to unravel its signaling pathways and potential therapeutic avenues.

#### 2.2.3 Receptor-interacting protein kinase 3

RIP3 is a serine/threonine kinase that plays a vital role in regulating programmed cell death, particularly necroptosis. High levels of RIP3 expression lead cells toward necroptosis, while lower levels favor apoptosis. Additionally, RIP3 promotes the release of IL-1β through the NLRP3-caspase-1 pathway (Frank and Vince, 2019). These findings suggest that RIP3 is crucial in balancing pyroptosis, apoptosis, and necroptosis. RIP3 is activated by the phosphorylation of RIP1, which facilitates the recruitment of other proteins necessary for necroptosis—a form of inflammatory cell death characterized by cell swelling and membrane rupture. This process differs from apoptosis and is associated with various pathological conditions, including neurodegenerative diseases, ischemic injury, and certain inflammatory disorders.

#### 2.2.4 Inflammasome

Inflammasomes, intricate multiprotein complexes, are central in regulating PANoptosis, a comprehensive cell death process (Oh et al., 2023). These assemblies consist of pattern recognition receptors (PRRs), an adapter protein, and caspase-1, which, when activated by pathogen-associated molecular patterns (PAMPs) and damage-associated molecular patterns (DAMPs), initiate pyroptosis. Activated caspase-1 cleaves and releases pro-inflammatory cytokines IL-1β and IL-18, as well as gasdermin D (GSDMD), facilitating pyroptotic cell death. In the context of PANoptosis, inflammasomes act as key integrators, enabling communication between pyroptosis and other cell death pathways. The activation of caspase-1, along with various effectors, orchestrates the simultaneous activation of multiple death mechanisms, resulting in PANoptosis.

There are five identified inflammasome sensors: NLRP1, NLRP3, absent in melanoma 2 (AIM2), NLRC4, and Pyrin, with the latter four playing direct roles in PANoptosis. NLRP3 activation and GSDMD-mediated pyroptosis are particularly important in the relationship between apoptosis and necroptosis. Upon activation of the NLRP3 inflammasome, inflammatory cells engage in PANoptosis. Although the knockdown of NLRP3 or GSDMD reduces early cell death, the overall incidence of inflammatory cell death rises over time, driven by caspase-8 and RIP3. This suggests that in the absence of NLRP3 or pyroptosis, cell death becomes dependent on caspase-8 and RIP3 (Zheng et al., 2020). During *P. aeruginosa* infection, the lack of NLRC4 results in the activation of alternative cell death pathways involving RIP1 and MLKL, while the activation of caspases 1, 3, 7, and 8 is reduced. This highlights the critical role of NLRC4 in the PANoptosis process (Sundaram et al., 2022).

Overall, inflammasomes are essential regulators of PANoptosis, with their orchestration of pyroptosis being vital for maintaining immune balance and influencing pathological conditions. Ongoing research is focused on elucidating their complex mechanisms and exploring their therapeutic potential in the context of PANoptosis.

## 3 PANoptosis in IS

**Figure 1:**
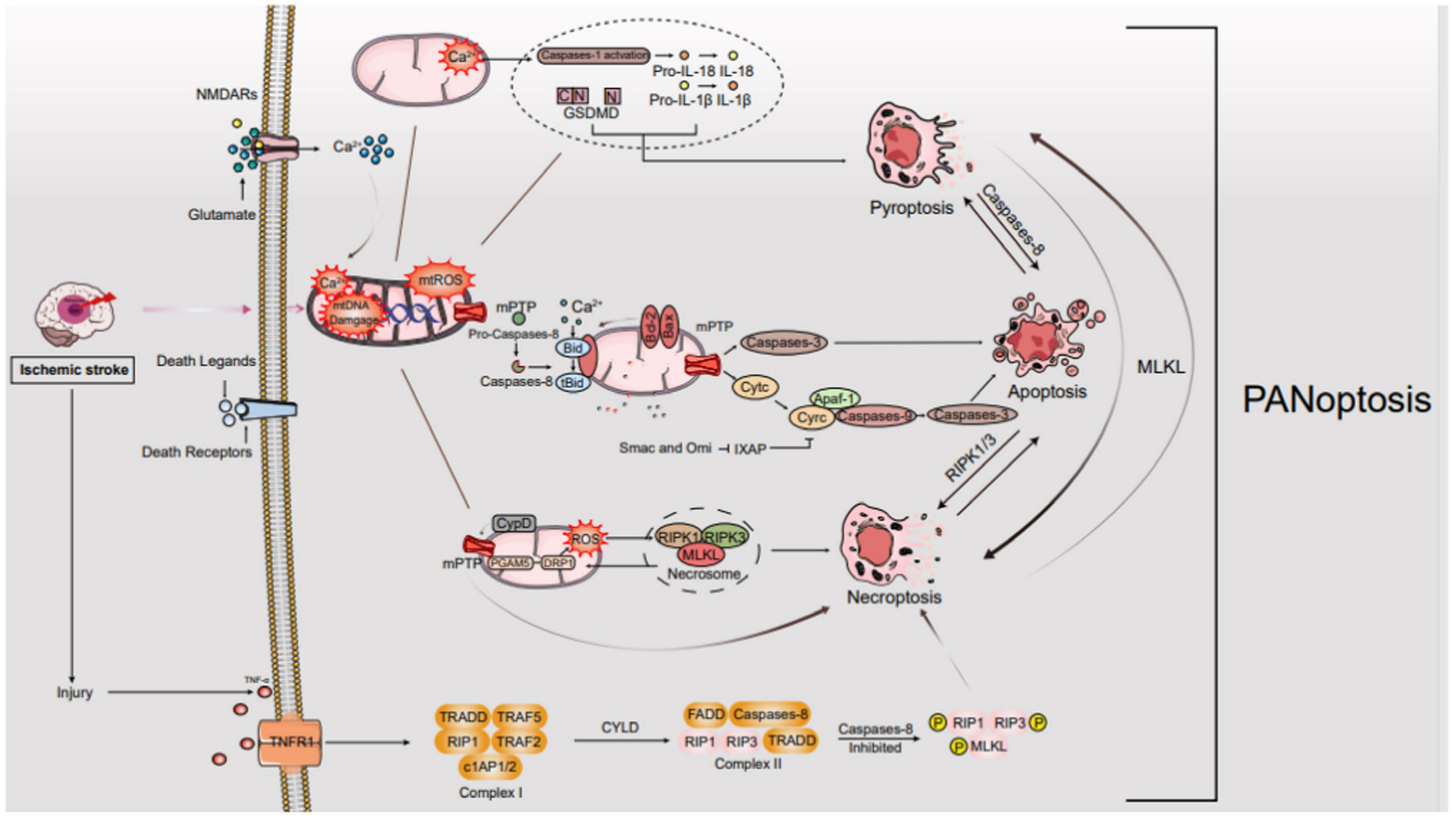
The mechanisms of PANoptosis in IS. In ischemic stroke, oxygen–glucose deprivation sparks a cascade leading to programmed cell death, notably PANoptosis. It starts with ATP depletion and death ligand binding to membrane receptors. As ischemia intensifies, Na^+^/K^+^ ATPase failure causes neuronal depolarization, unleashing glutamate, ROS, free radical damage, Ca^2+^ imbalance, and EAA toxicity. Death ligand-receptor interactions trigger signaling cascades that selectively engage FADD for apoptosis or TRADD for necroptosis. These cascades disrupt mitochondria, manifesting as mPTP formation, mtROS surge, Ca^2+^ overload, and mtDNA damage. These mitochondrial perturbations are crucial in orchestrating diverse cell death pathways, encompassing apoptosis, necroptosis, and possibly other PANoptosis modalities.

PANoptosis, a term coined to encompass a broader spectrum of cell death modalities beyond the classical apoptosis, autophagy-dependent cell death, and necroptosis, refers to a concept that encompasses multiple regulated cell death pathways that can be triggered simultaneously or sequentially under certain stress conditions. This holistic view of cell death recognizes that cells, particularly in response to severe insults or pathological states like ischemic stroke, may engage in a complex interplay of molecular mechanisms leading to their demise. In the context of ischemic stroke, a sudden interruption of blood flow to a part of the brain, PANoptosis becomes particularly relevant as it highlights the intricate interplay of various cell death mechanisms that contribute to tissue damage and neurological dysfunction. Ischemic stroke triggers a cascade of events, including energy depletion, oxidative stress, excitotoxicity, and inflammation, which ultimately lead to neuronal and glial cell death (Figure 1).

The mechanisms of PANoptosis in IS. In ischemic stroke, oxygen–glucose deprivation sparks a cascade leading to programmed cell death, notably PANoptosis. It starts with ATP depletion and death ligand binding to membrane receptors. As ischemia intensifies, Na^+^/K^+^ ATPase failure causes neuronal depolarization, unleashing glutamate, ROS, free radical damage, Ca^2+^ imbalance, and EAA toxicity. Death ligand-receptor interactions trigger signaling cascades that selectively engage FADD for apoptosis or TRADD for necroptosis. These cascades disrupt mitochondria, manifesting as mPTP formation, mtROS surge, Ca^2+^ overload, and mtDNA damage. These mitochondrial perturbations are crucial in orchestrating diverse cell death pathways, encompassing apoptosis, necroptosis, and possibly other PANoptosis modalities.

Recent discoveries on PCD pathways’ intricate interplay reveal multifaceted signaling platforms. Pyroptosis, apoptosis, and necroptosis often coexist in cellular stress, contributing to neuroinflammation with health implications. PANoptosis’s components linked to neurological disorders underscore the potential of unraveling its mechanisms for innovative therapies (Malireddi et al., 2020). PANoptosis is often associated with inflammatory reactions in various central nervous system (CNS) diseases (Lunemann et al., 2021; McKenzie et al., 2020; Yan et al., 2021). Core components of the PANoptosome, such as the inflammasome, caspase-8, RIPK1, and others, have been linked to neuronal death in various contexts (Degterev et al., 2019; Fricker et al., 2018). Inflammation and activation of the immune system are frequently implicated in the pathophysiology of IS, which can result in severe brain damage (Zhang F. et al., 2022). Existing studies on PANoptosis have observed similarities in the expression of cell death and the underlying pathophysiological mechanisms related to inflammation in IS. These findings provide fundamental evidence supporting the potential existence of PANoptosis and PANoptosomes (Yan et al., 2022). Furthermore, it has been reported that glial cells can modulate these three forms of cell death following injury, which aligns with the findings in existing studies of PANoptosis that are related to inflammation and the immune system (Liu X. et al., 2022). Additionally, research has indicated that certain molecules can concurrently affect two components of PANoptosis during IS. Notably, RIPK3, a key mediator of necroptosis, engages in crosstalk with the Jun N-terminal kinase-driven inflammatory signaling cascade, which is intimately tied to neuronal apoptosis processes (Hu et al., 2020). Inhibiting thromboxane A synthase/thromboxane A2/thromboxane prostanoid signaling has been demonstrated to concurrently suppress both apoptosis and pyroptosis (Chueh et al., 2020). Furthermore, the nucleotide oligomerization domain-like receptors with caspase activation and recruitment domain 4 (NLRC4) inflammasome complex has the capacity to simultaneously modulate both apoptosis and pyroptosis (Poh et al., 2019). Therefore, it is possible to simultaneously regulate and intervene in PANoptosis induced by IS.

Although PANoptosome’s direct role in IS is unexplored, its components are abundant in the brain. Research shows inhibiting TAK1 reduces neuronal death from these conditions, hinting at PANoptosome’s potential involvement (Wang J. X. et al., 2019; Wu W. X. et al., 2020). Moreover, TAK1 plays a crucial role in modulating microglial function and its interaction with an inflammatory pathway that triggers neuronal apoptosis and pyroptosis (Zeyen et al., 2020). Furthermore, it plays a critical role in the crosstalk between necroptosis and apoptosis in neurons during IS (Naito et al., 2020). These findings hint at TAK1’s regulatory role in PANoptosomes during IS. Yan et al.’s study shows PANoptosis-like death in R28 retinal cells exposed to oxygen–glucose deprivation/recovery, a model mimicking ischemia–reperfusion. Combined inhibitors targeting apoptosis, pyroptosis, and necroptosis effectively reduced cell death, especially when combined, confirming PANoptosis’s presence in R28 cells (Yan et al., 2023).

## 4 Autophagy and its mechanism

### 4.1 Autophagy overview

Introduced by Christian de Duve in 1963, “autophagy” stems from Greek roots for “self-eating.” (De Duve and Wattiaux, 1966). This conserved mechanism in eukaryotes maintains cellular balance by degrading organelles, proteins, lipids, and nucleic acids, recycling their components, especially during nutrient deprivation. Autophagy also eliminates pathogens, damaged organelles, and abnormal proteins. Dysregulation of autophagy is associated with diseases like neurodegeneration, infections, inflammation, metabolic disorders, and aging (Dikic and Elazar, 2018).

Autophagy falls into two main types: bulk/non-selective and selective. Bulk autophagy degrades various cytoplasmic components indiscriminately. In contrast, selective autophagy targets specific cargos, often those posing threats or requiring removal. It encompasses diverse pathways, each targeting distinct cellular components like mitochondria, lysosomes, protein/RNA aggregates, pathogens, ER, peroxisomes, ribosomes, ferritin, glycogen, lipid droplets, and fluid. Effective degradation of these cargos is crucial for physiological functions, and failure can lead to diseases. These cargos are intricately tied to cellular health and function (Jiang and Mizushima, 2014). Both autophagy types involve autophagosome formation, spherical double-membranes engulfing cellular content for degradation. This process is tightly regulated by Atg proteins, conserved from yeast to mammals. Over 40 Atg genes exist, with core Atg1-18 crucial for both autophagy types. These proteins orchestrate autophagosome formation and function, vital for maintaining cellular homeostasis and eliminating unwanted/damaged components.

### 4.2 Summary of autophagy main proteins

Over 30 autophagy-related (Atg) proteins participate in autophagy, categorized into four key functional groups: (1) Initiation Complex: This comprises unc-51-like kinase 1 (ULK1), Atg13, and Atg17, which collaboratively initiate the autophagy process. (2) Class III PI3K Complex for Autophagy Activation: Constituted by phosphatidylinositol 3-kinase (PI3K), Beclin1, Atg14, and AMBRA1, this complex fosters phagophore formation, a crucial step in autophagy. (3) Atg12 Conjugation System: Involving Atg12, Atg5, Atg16L1, and Atg10, this system orchestrates the conjugation reactions essential for autophagy progression. (4) The LC3 (Light Chain 3) conjugation and lipidation system:LC3 exists as LC3-I (cytosolic, inactive) and LC3-II (lipidated, active). The conversion from LC3-I to LC3-II involves activation by ATG7, conjugation with ATG3, and lipidation by the ATG5-ATG12 complex. LC3B-I levels indicate autophagy activity, while LC3B-II marks autophagosome formation and interacts with autophagy receptors for selective substrate degradation (Yoshii and Noboru, 2017). LC3-II and p62 (SQSTM1) are autophagy markers. LC3-II decorates autophagosome membranes, while p62 tags cargo for degradation. High LC3-II levels (more autophagosomes) and low p62 (efficient cargo degradation) signify increased autophagy flux. Flux covers autophagosome formation, fusion with lysosomes, and cargo degradation. Fusion disruption or lysosomal dysfunction elevates both markers. Thus, autophagy studies should employ multiple markers and techniques for clarity (Klionsky et al., 2021).

### 4.3 Signaling pathways of autophagy in stroke

#### 4.3.1 Mammalian target of rapamycin signaling pathway

Multiple signaling pathways, responsive to both internal and external cues, coordinate autophagy. Here, we focus on pathways that may explain gender-specific autophagy differences in stroke (Martens, 2018). mTOR, a serine/threonine kinase, dually regulates autophagy via ULK1 phosphorylation. Central to cell processes, mTOR comprises mTORC1 and mTORC2. Rapamycin-sensitive mTORC1 drives anabolism and inhibits autophagy, especially in the brain. Rapamycin-insensitive mTORC2 contributes to Akt activation and diverse signaling pathways (Shi et al., 2019). Asiaticoside, in a rat model of dementia, effectively diminishes autophagic activity and enhances memory performance by regulating the mTOR signaling pathway (Guo et al., 2021). During ischemia and reperfusion processes, mTOR plays a crucial role in the regulation of autophagy through two primary pathways: the AMPK-mTOR pathway and the Akt–mTOR pathway (Wang et al., 2020).

The 5′-AMP-activated protein kinase (AMPK) is a serine/threonine kinase that becomes activated in response to a decrease in the ATP/AMP ratio, a condition often observed after an IS. AMPK exerts its effect by inhibiting mTOR, leading to the subsequent activation of autophagy (Ke et al., 2018). Once activated, AMPK initiates the activation of the ULK1 complex, which subsequently undergoes autophosphorylation. This autophosphorylation event is essential for phosphorylating specific autophagy-initiating protein complexes, thereby initiating the process of autophagy (Thapa et al., 2021). Autophagy, which is mediated by AMPK, plays a crucial role in the neuroprotection afforded by ischemia preconditioning. This suggests that AMPK holds promise as a potential therapeutic target for IS. Evidence further indicates that the AMPK-mTOR signaling pathway effectively regulates autophagy activation through the coordinated phosphorylation of ULK1 (Wang Z. et al., 2018). Simultaneously, PI3K enzymes play a role in the modulation of the Akt–mTOR pathway. PI3K serves as a critical regulator of autophagy and is involved in phagosome maturation. There are three main classes of PI3K: Class I, II, and III. Class III PI3K(Vps34), takes part in complex formation with beclin-1, contributing to the regulation of autophagy. In contrast, class I PI3Ks are primarily involved in the activation of Akt (Noorolyai et al., 2019).

#### 4.3.2 Mitogen-activated protein kinase signaling pathway

The MAPK signaling pathway comprises p38 extracellular regulated protein kinases (p38-ERK), ERK, and C-Jun N-terminal kinase (JNK) (Kim and Choi, 2015). Early stroke, p38 MAPK activation supports neuronal survival, anti-inflammation, and anti-apoptosis via Elk1, CHOP10, LEF2C, and MAPKK2/3. Later, p38 overactivation triggers target gene expression, activating transcription factors and caspases (Ferrer et al., 2003; Li et al., 2015; Song et al., 2016). indorsing neuronal apoptosis. It is evident that interventions for IS should be tailored to target specific p38 MAPK signal molecules. Notably, p38 inhibitors enhance cell survival routes, such as ERK, and alleviate ischemic mitochondrial fragmentation and autophagy. This, in turn, reduces the volume of ischemic cerebral infarction and preserves nerve function (Zhang et al., 2015). This evidence supports the notion that ERK, JNK, and p38 MAPK are involved in mediating autophagy in IS, with distinctive roles in modulating this process.

#### 4.3.3 Nuclear factor-kappa B pathway

NF-κB, a transcription factor, governs the expression of numerous genes (Chen et al., 2011). In ischemic conditions, IκappaB kinase (IKK) becomes activated and subsequently phosphorylates IκBa. This leads to the release of NF-κB, which is then translocated into the nucleus to facilitate autophagy (Jiang et al., 2012). Notably, in mouse models, the knockout of the NF-κB p50 subunit has been observed to promote autophagy by inhibiting the mTOR pathway after cerebral ischemia (Li et al., 2013). This insight sheds light on the complex regulatory mechanisms at play during ischemic conditions and their impact on autophagy processes. Also, it has been demonstrated that the NF-κB-dependent p53 signaling pathway is involved in the cellular response after ischemic events (Cui et al., 2013). In the context of brain ischemia/reperfusion, it has been observed that p53-dependent nuclear factor NF-κB expression is induced. Simultaneously, the damage-regulated autophagy modulator (DRAM) serves as a crucial regulator of p53-dependent autophagy. This DRAM-mediated NF-κB/p53 pathway plays an active role during the ischemia/reperfusion process, contributing to both apoptosis and autophagic cell death (Gupta et al., 2019). Moreover, it’s worth noting that autophagy and apoptotic pathways may interplay to influence programmed cell death by modulating the p53 pathway. This complex interplay suggests that targeting autophagy via mTOR to mediate the NF-κB-p53 signaling molecule could offer a promising approach for stroke treatment (Sun B. et al., 2018).

#### 4.3.4 Hypoxia-inducible factor-1 pathway

HIF-1α, activated under hypoxia, regulates autophagy post-stroke. It’s a heterodimer with HIF-1β. HIF-1α targets BNIP3, which triggers autophagy by displacing Bcl-2 from beclin-1 (Mo et al., 2020). Recent reports indicate that Hydroxysafflor Yellow A exerts neuroprotective effects by activating neuronal autophagy after oxygen–glucose deprivation/reoxygenation (OGD/R) through the HIF-1α/BNIP3 pathway (Wei et al., 2022). Furthermore, there are indications that BNIP3L/NIX is implicated in mitochondrial autophagy induced by cerebral ischemia–reperfusion, suggesting that BNIP3L could be a potential therapeutic target for addressing IS (Yuan et al., 2017). BNIP3 also has the capacity to inhibit Rheb, which results in the reduction of mTOR activity and the subsequent initiation of autophagy. Nevertheless, the expression of HIF-1 is associated with the upregulation of mitochondrial autophagy through mTOR pathway inhibition. However, further verification is required to confirm whether this effect is mediated by BNIP3 (Sun Y. et al., 2018).

#### 4.3.5 Peroxisome proliferator-activated receptor

PPAR-γ, a member of the hormone receptor superfamily, functions as a ligand-activated transcription factor. PPARγ agonists have demonstrated anti-inflammatory and antioxidant properties in stroke (Li Q. et al., 2019). Additionally, Li and colleagues revealed that luteoloside, a flavonoid, significantly suppressed the activation of the NF-κB signaling, elevated PPARγ protein expression, and enhanced nuclear accumulation of Nrf2 in rats subjected to MCAO. Furthermore, the anti-inflammatory impact of luteoloside was closely associated with the actions of both PPARγ and Nrf2 (Li Q. et al., 2019). Furthermore, Zhao and colleagues concluded that Berberine, an alkaloid compound derived from herbs, plays a beneficial role in providing neuroprotection against cerebral ischemia/reperfusion (I/R) injury. It achieves this by up-regulating PPAR-γ to inhibit NF-κB-mediated pyroptosis (Zhao et al., 2021).

#### 4.3.6 Unfolded protein response pathway

The UPR, an intracellular signaling cascade, responds to unfolded/misfolded ER proteins. In cerebral ischemia, it’s a double-edged sword, initially promoting survival but potentially causing cell death with persistent activation. Regulated by ATF6, IRE1, and PERK, these sensors detect ER stress, triggering signaling for protein folding, degradation, and ER biogenesis gene activation (Xiaowei et al., 2023). This coordinated response aims to restore ER function and mitigate the damage caused by ischemia. However, the precise regulation of the UPR in this context is complex, involving crosstalk with other signaling pathways and feedback loops that fine-tune its activation.

The UPR’s effects in cerebral ischemia vary, depending on context. Initially, it promotes survival by enhancing protein folding and reducing synthesis. However, prolonged activation may induce apoptosis and necrosis, worsening ischemia damage. The balance between protective and harmful effects is influenced by ischemia severity, duration, and cellular/molecular environment.

#### 4.3.7 Reactive oxygen species pathway

The ROS pathway, critical in cellular metabolism and signaling, produces signaling molecules under normal conditions, regulating growth, differentiation, and apoptosis. Oxidative stress arises when ROS outpaces antioxidant defenses, damaging cells. Mitochondria are primary ROS sources, with contributions from other compartments. NADPH oxidases and nitric oxide synthases facilitate ROS production. In cerebral ischemia, ROS have dual roles: signaling for adaptation and survival, but excessive levels cause oxidative stress, damaging proteins, lipids, and DNA. This damage triggers cell death pathways, exacerbating neuronal injury and stroke pathophysiology.

## 5 Autophagy in IS

**Figure 2:**
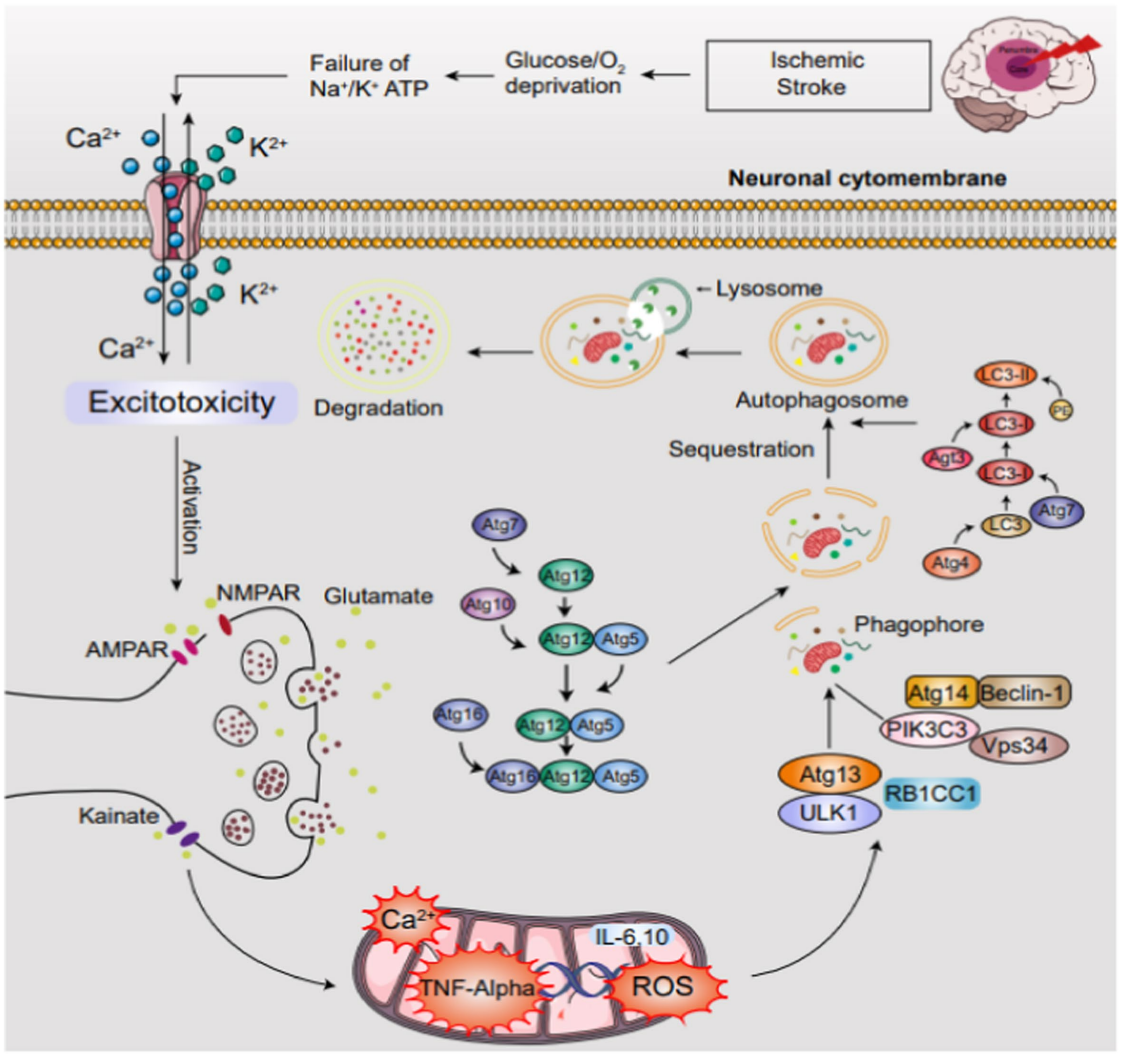
The mechanisms of autophagy in IS. Under ischemic conditions, autophagy is orchestrated by the ULK1 and PtdIns3K complexes, facilitated by Atg genes. The process unfolds in phases: induction initiates phagophore formation, which matures into a double-membraned autophagosome. Fusion.

Cerebral ischemia, characterized by inadequate brain blood flow, causes neuronal damage and dysfunction. Reperfusion, vital for tissue survival, paradoxically triggers Cerebral Ischemia–Reperfusion Injury (CIRI). Autophagy, a cellular self-cleaning process, has garnered attention in neurology due to its dual roles in these contexts. Autophagy, a tightly regulated process, degrades and recycles cellular components to maintain homeostasis. Early in ischemia, autophagy activates as a neuroprotective response, eliminating damaged proteins and organelles. However, prolonged ischemia and CIRI during reperfusion can lead to excessive autophagy, degrading essential components and exacerbating brain damage (Figure 2).Thus, autophagy’s role in ischemic stroke is intricate, influenced by context, timing, and intensity. Researchers are investigating autophagy modulation as a potential therapeutic strategy. Pharmacological interventions targeting autophagy could enhance neuroprotection during ischemia’s acute phase or mitigate neuronal injury during reperfusion.

The mechanisms of autophagy in IS. Under ischemic conditions, autophagy is orchestrated by the ULK1 and PtdIns3K complexes, facilitated by Atg genes. The process unfolds in phases: induction initiates phagophore formation, which matures into a double-membraned autophagosome. Fusion with lysosomes forms an autolysosome, where lysosomal enzymes degrade its contents, completing the autophagy cycle.

### 5.1 Role of autophagy in IS

Autophagy holds a pivotal role in ischemic stroke’s pathological and physiological processes, engaging various pathways. Multiple studies underscore its activation as a response to ischemia/reperfusion injury, emphasizing its complexity and significance in stroke management (Hou et al., 2019). Autophagy activation was confirmed through electron microscopy, where an increased number of autophagosomes were observed. Specifically, in mice subjected to middle cerebral artery occlusion (MCAO) followed by reperfusion, elevated levels of LC3 protein were detected in the ischemic penumbra (Nitatori et al., 1995). The research findings indicate that following 1 h of ischemia, there was a significant increase in LC3 expression within the ischemic penumbra. This heightened expression persisted for a duration of 5 h in the absence of reperfusion (Yan et al., 2011). Meaningfully, the presence of cleaved caspase-3 was detected, and its temporal pattern mirrored that of autophagy. This observation suggests that both autophagy and apoptosis coexist within the cerebral ischemic penumbra (Sodhi et al., 2021). However, the question of whether autophagy activation promotes neuronal survival or leads to cell death remains a subject of debate. Some researchers argue that autophagy induction following a stroke serves to supply the cell with energy by removing damaged cellular components. On the contrary, there is an opposing viewpoint suggesting that excessive autophagy can worsen stroke injury. This is because it can lead to the destruction of healthy cells and trigger autophagic cell death, in addition to apoptosis (Wang, et al., 2020). Hence, evaluating the timing and extent of autophagy’s induction or modulation can elucidate its role in cerebral ischemia. While additional mechanistic insights into the autophagy pathway are crucial, it emerges as a promising therapeutic target for stroke. Based on experimental evidence, two opposing perspectives emerge regarding autophagy’s potential influence on ischemic neurons (Hou et al., 2019).

### 5.2 Autophagy beneficially affects in IS

Following ischemia and subsequent reperfusion, a range of environmental disruptions, including ER stress, oxidative stress, mitochondrial dysfunction, and cellular death, have the potential to exacerbate neurological dysfunction (Khan et al., 2020). It has been reported that Lomitapide demonstrated significant benefits in terms of enhancing survival rates, decreasing neuronal tissue loss, and improving neurological function following MCAO. Moreover, lomitapide exhibited the capacity to enhance LC3-II expression, decrease P62 and LAMP2 expression, thereby facilitating autophagic flux. Additionally, it suppressed apoptosis by upregulating the expression of the anti-apoptotic protein Bcl-2 and downregulating the expression of the pro-apoptotic protein Bax. This suggests that Lomitapide facilitates autophagy (Zheng et al., 2022). Furthermore, Zhang et al. reported that astragaloside IV has the potential to reduce neuronal apoptosis in HT22 cells following OGD/R (oxygen–glucose deprivation and reoxygenation) by stimulating autophagy (Zhang D. M. et al., 2019). Additionally, Ezetimibe has demonstrated its efficacy in reducing infarct volume and improving neurobehavioral deficits in rats subjected to MCAO. However, the neuroprotective and antiapoptotic benefits of ezetimibe were found to diminish when an autophagy inhibitor, 3-MA, was introduced as an intervention (Jing et al., 2018). These studies indicate that the activation of autophagy in response to ischemia and reperfusion may have a neuroprotective effect by reducing brain damage through the inhibition of apoptosis. The study has demonstrated that rapamycin, an inhibitor of mTOR known for its role in regulating energy balance and suppressing autophagy, effectively reduces infarction volumes and enhances neurological outcomes (Wu et al., 2018). This protective effect of rapamycin has been consistently observed in both clinical and pre-clinical studies involving IS. To delve into the specifics, the restoration of autophagy, achieved by increasing NAD^+^: NADH levels or elevating Beclin1 expression, has shown significant benefits. These interventions improved mitochondrial function and contributed to a reduction in stroke development, particularly in stroke-prone spontaneously hypertensive rats (Forte et al., 2020). In addition, the overexpression of BAG3 triggers autophagy activation while concurrently suppressing apoptosis, thus serving as a potential therapeutic strategy for mitigating IS and hypoxia/reoxygenation injuries. This suggests a promising therapeutic role for BAG3 expression in cerebral ischemia (Liu M. et al., 2023). Also, Wang and his colleagues confirmed that the long non-coding RNA (lncRNA) Metastasis-associated lung adenocarcinoma transcript 1 (MALAT1) additionally triggers autophagy and offers protection against cerebral ischemia. This protective effect is achieved through its interaction with miR-200c-3p, leading to the upregulation of Sirt1 expression (Wang L. et al., 2019).

### 5.3 Negative effect of autophagy in IS

Accumulated evidence indicates that autophagy activation may worsen neurological dysfunction post-ischemia/reperfusion. During ischemia, a sophisticated interplay of interconnected pathways initiates autophagy, highlighting its intricate involvement (Wang et al., 2020). One such example is the association observed between elevated LC3-II levels (indicative of an increased number of autophagosomes) and the extent of cortical infarction in rat models subjected to MCAO (Acaz-Fonseca et al., 2020).

The administration of tetrahydroxystilbene glucoside (TSG), a constituent of *Fallopia multiflora*, similarly reduced infarct volume and improved neurobehavioral deficits in ischemia/reperfusion mice by suppressing autophagy (Yu et al., 2019). The study further illuminates TIGAR’s (TP53-induced glycolysis and apoptosis regulator) role as a fructose-2,6-bisphosphatase in modulating autophagy post-ischemia/reperfusion. TIGAR-transgenic mice exhibited reduced autophagy, whereas TIGAR-knockout mice showed enhanced autophagy, accompanied by larger infarcts and heightened neurological deficits. Notably, 3-MA administration post-ischemia/reperfusion mitigated these adverse effects, suggesting TIGAR’s neuroprotective role partly stems from inhibiting autophagy (Zhang Y. et al., 2019). Puerarin, a traditional Chinese herb, has been shown to mitigate brain dysfunction following ischemia/reperfusion by inhibiting the expression of autophagy proteins through modulation of the AMPK-mTOR-ULK1 signaling pathway (Wang J. F. et al., 2018). Correspondingly, Liu and his colleagues confirmed that Activin A enhanced neurological function and decreased infarct size in mice afflicted by MCAO/R-induced IS through the inhibition of autophagy (Liu X. et al., 2023). Moreover, Calycosin alleviates CIRI by attenuating autophagy through the STAT3/FOXO3a signaling pathway (Xu et al., 2023). Zhang et al. have uncovered that Danhong injection mitigates cerebral ischemia–reperfusion injury by restraining autophagy via the miRNA-132-3p/ATG12 signaling axis (Zhang H. et al., 2023). These findings indicate that suppressing autophagy provides neuroprotection.

Following an IS, the activation of autophagy remains a subject of ongoing debate. The presence of autophagy in cells undergoing ischemic injury at different stages raises the question of whether it plays a role in neuroprotection or contributes to apoptosis (Hou et al., 2019).

## 6 Interplay between PANoptosis and autophagy in IS

PANoptosis and autophagy are different forms of cell death, but numerous lines of structural, functional, and mechanical evidence show that crosstalk ensues between them.

### 6.1 The interplay of autophagy and apoptosis in IS

**Figure 3:**
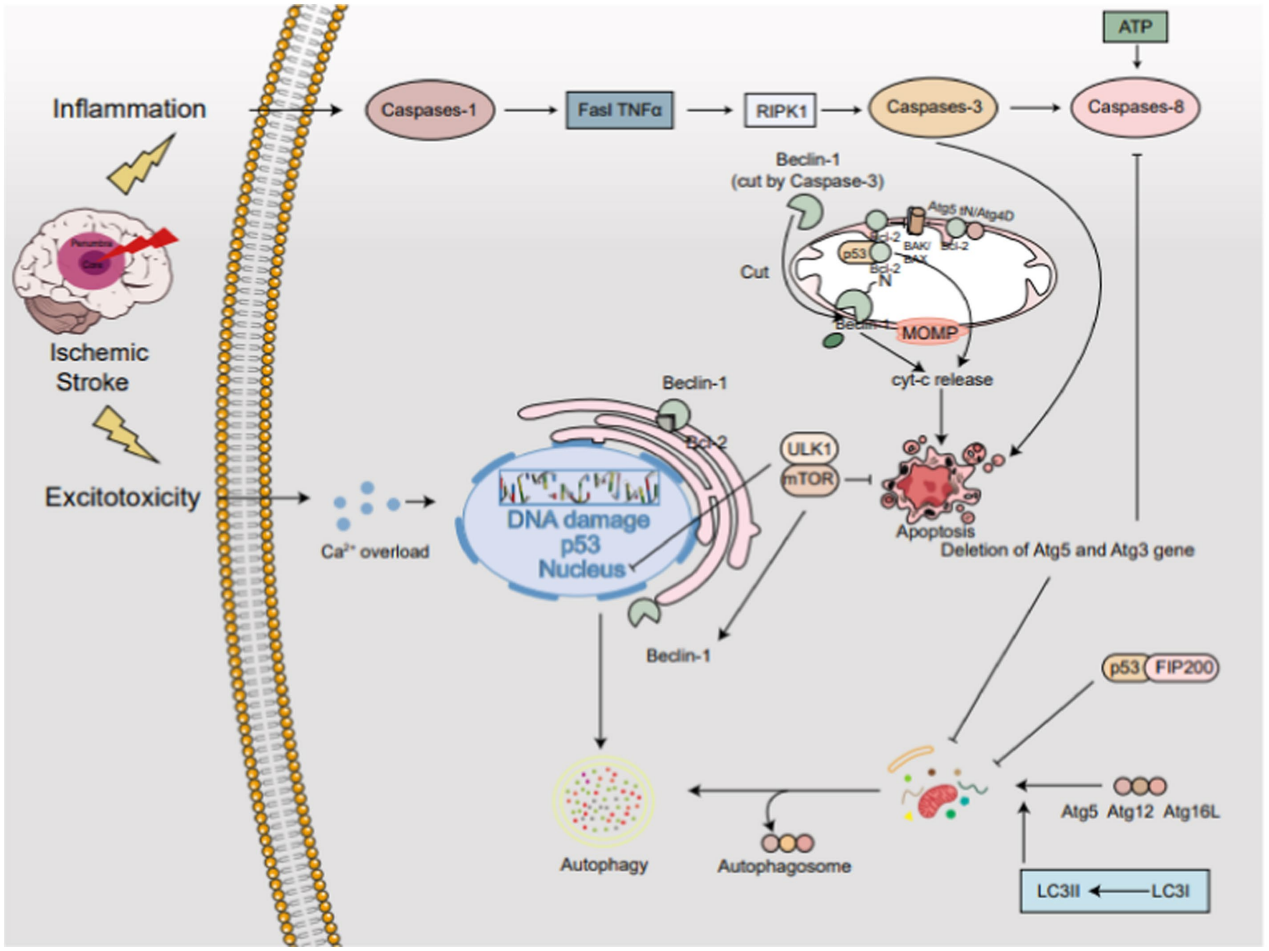
The interplay between autophagy and apoptosis in IS. The PI3K/AKT/mTOR axis serving as a central hub. Beclin-1, residing in the endoplasmic reticulum, acts as a bifunctional player, promoting autophagy independently but inhibiting it when bound to Bcl-2. Caspase-3-mediated cleavage of Beclin-1 redirects it to mitochondria, triggering apoptosis. The Atg5-Atg12-Atg16L complex activates LC3-I to LC3-II, pivotal for autophagy progression. Conversely, calpain-cleaved Atg5tN enters mitochondria, interacting with BCL-2 proteins to unleash Cyt-c and foster apoptosis. Atg4D variants further fine-tune the autophagy-apoptosis balance. p53 exhibits a dual-edged sword, promoting apoptosis by associating with Bcl-2 in mitochondria but enhancing autophagy via the mTOR pathway in the nucleus. In the cytoplasm, p53 engages with FIP200 to restrain autophagy. Notably, ATG5 and ATG3 deletions impede both autophagy and apoptosis, whereas caspase-3-mediated Beclin-1 cleavage propels apoptosis.

Autophagy and apoptosis, two fundamental cellular processes, govern cell fate under stress. Recently, attention has shifted towards dual regulators that interface between these two pathways. Despite observed interactions among autophagy- and apoptosis-related proteins, their underlying regulatory mechanisms remain elusive. Notably, autophagy and apoptosis display a dual role, exhibiting both protective and detrimental effects at distinct stages of IS progression (Figure 3).

The interplay between autophagy and apoptosis in IS. The PI3K/AKT/mTOR axis serving as a central hub. Beclin-1, residing in the endoplasmic reticulum, acts as a bifunctional player, promoting autophagy independently but inhibiting it when bound to Bcl-2. Caspase-3-mediated cleavage of Beclin-1 redirects it to mitochondria, triggering apoptosis. The Atg5-Atg12-Atg16L complex activates LC3-I to LC3-II, pivotal for autophagy progression. Conversely, calpain-cleaved Atg5tN enters mitochondria, interacting with BCL-2 proteins to unleash Cyt-c and foster apoptosis. Atg4D variants further fine-tune the autophagy-apoptosis balance. p53 exhibits a dual-edged sword, promoting apoptosis by associating with Bcl-2 in mitochondria but enhancing autophagy via the mTOR pathway in the nucleus. In the cytoplasm, p53 engages with FIP200 to restrain autophagy. Notably, ATG5 and ATG3 deletions impede both autophagy and apoptosis, whereas caspase-3-mediated Beclin-1 cleavage propels apoptosis.

#### 6.1.1 mTOR

The PI3K/AKT/mTOR pathway, a quintessential signaling cascade, functions to bolster cell viability, deter apoptosis, and impede autophagy. In an environment with sufficient nutrients, signals like growth factors, glucose, and amino acids engage with the serine/threonine phosphorylation-mediated mTOR complex 1 (mTORC1) signaling pathway, resulting in the suppression of autophagy (Russell et al., 2014). In IS, mTOR activation is bifurcated: it supports cell survival via protein synthesis and metabolism, yet unchecked activity may drive dysfunction, contributing to stroke pathogenesis. mTOR centrally regulates autophagy, and under ischemia, its inhibition triggers autophagy. The intricate balance of mTOR-mediated pathways intersects multiple cellular processes. Intriguingly, inhibiting mTOR has shown potential to mitigate apoptosis and necroptosis in ischemia-mimicking cellular models (Jiang et al., 2024). This finding suggests that modulating mTOR activity might hold promise as a therapeutic strategy to mitigate apoptosis in stroke. Conversely, excessive mTOR activation could promote apoptosis by enhancing cellular stress and dysfunction.

#### 6.1.2 Beclin-1

Beclin-1, a vital regulatory protein in autophagosome formation, performs multiple functions. Free Beclin-1 enhances autophagy, while its binding to Bcl-2 suppresses it (Li Z. G. et al., 2019). As a novel Caspase substrate, Beclin-1 is cleaved by Caspases 3–10, losing its autophagy-inducing capability. The cleaved C-terminal fragment relocates to mitochondria, triggering Cyt-c release and apoptosis. Thus, Beclin-1 plays a dual role in apoptosis and autophagy.

#### 6.1.3 Anti-thymocyte globulin

Current findings emphasize Atg’s pivotal regulatory function in autophagy and apoptosis. Early in autophagosome genesis, the Atg12-Atg5-Atg16L complex catalyzes LC3-I to LC3-II, which integrates into the autophagosome membrane, facilitating its expansion. Beyond complex formation, Atg proteins modulate autophagy through alternate pathways. Additionally, Atg proteins regulate autophagy via complex assembly and apoptosis by cleaving specific targets. In apoptosis, calpain-mediated cleavage of non-conjugated Atg5 releases Atg5tN, which enters mitochondria and interacts with Bcl-2 proteins to release cytochrome c. Notably, Atg5 knockdown in mice subjected to MCAO-induced ischemia increased beclin1 and LC3-II expression, suggesting attenuated autophagy-mediated ferroptosis (Zhu et al., 2024). In addition, research has corroborated that indobufen exerts protective effects against OGD/R-induced injury in SH-SY5Y cells by inhibiting autophagy, oxidative stress, and apoptosis. This protective mechanism involves the modulation of the transcription factor NRF2 and the suppression of Atg5 expression, thereby mitigating IS damage (Wang R. et al., 2024).

#### 6.1.4 Caspase

Atg3 is crucial for autophagosome biogenesis, featuring a Caspase-8 cleavage site within its sequence. Introducing mutations to this site significantly inhibits Caspase-8 proteolytic activity *in vitro*. Studies reveal that the absence of Atg5 or Atg3 disrupts autophagosome formation, thereby suppressing Caspase-8 activation and apoptosis. Similarly, Caspase-3 can cleave autophagy-related proteins, with Beclin-1 harboring two such sites, implicating its role in regulating autophagy and apoptosis. Following Caspase-3 cleavage, the unveiled BH3 domain of Beclin-1 may intensify apoptosis by engaging with anti-apoptotic Bcl-2 family members. Furthermore, research indicates that morphine preconditioning mitigates ischemia/reperfusion-induced Caspase 8-mediated neuronal apoptosis (Huang et al., 2024). In addition, As the study demonstrates, Astragaloside-IV suppressed the upregulation of Caspase-8 and Bax/Bcl-2 mRNAs, as well as the protein levels of apoptosis cytokines Caspase-8, cleaved Caspase-3, and Cyto C following ischemia reperfusion. This suggests that Astragaloside-IV mitigates ischemia reperfusion-induced apoptosis by inhibiting the activation of key factors in both the death receptor and mitochondrial pathways (Yin et al., 2020).

#### 6.1.5 P53

P53, a key apoptosis regulator, triggers apoptosis via two distinct mechanisms. Firstly, acting as a transcription factor, p53 can upregulate pro-apoptotic genes while suppressing anti-apoptotic genes. Additionally, p53 can relocate to the mitochondria and engage with Bcl-2 family proteins, resulting in mitochondrial membrane permeability and the release of Cyt-c (Castrogiovanni et al., 2018). Under normal conditions, p53 is typically found in the cytoplasm, but upon DNA damage, it is translocated to the nucleus. Recent research indicates that nuclear p53 promotes autophagy by modulating the mTOR pathway in a transcription-dependent manner (Denisenko et al., 2018). However, in the cytoplasm, p53 exhibits opposing effects, inhibiting autophagy by interacting with FIP200. This interaction prevents the activation of the ulk1-fip200-Atg13-Atg101 complex, thus impeding autophagosome formation. The research concludes that hyperglycemia can exacerbate brain injury stemming from I/R by potentially stimulating excessive autophagy. Additionally, it is postulated that hyperglycemia activates the intricate p53-Sesn2-AMPK signaling cascade, further contributing to the pathophysiological processes (Wang et al., 2024a).

### 6.2 The interplay of autophagy and pyroptosis in IS

**Figure 4:**
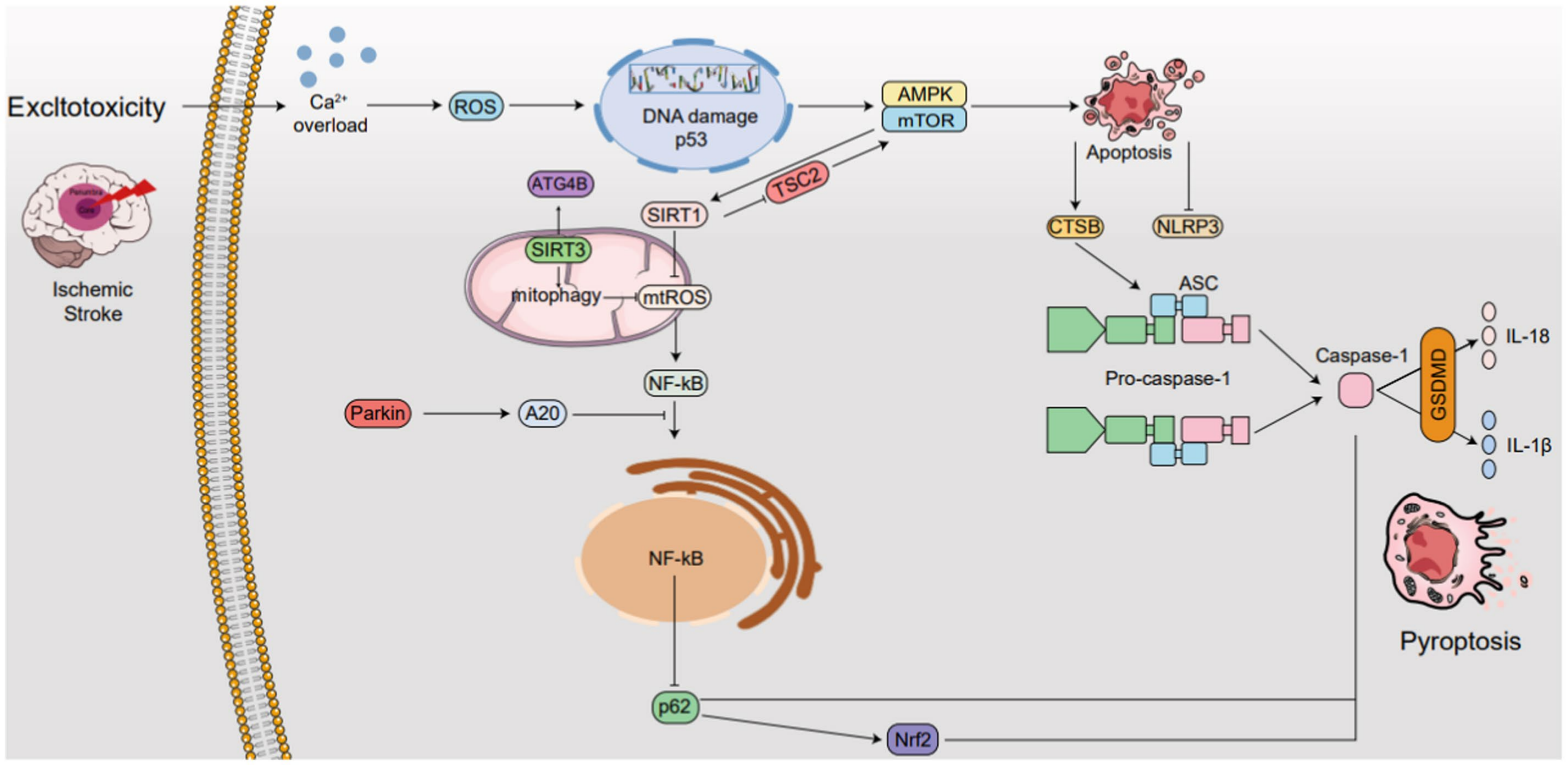
The interplay between autophagy and pyroptosis in IS. SIRT1 is not only induced by mTOR, it also induces mTOR by inhibiting TSC2 activity, while SIRT3 also activates ATG4B. SIRT1 suppresses ROS in mitochondria, while SIRT3 enhances mitophagy and also reduces ROS levels, thereby inhibiting NLRP3 inflammasome activation. Additionally, NLRP3 expression reciprocally regulates mitochondrial damage and ROS production. Autophagy boosts CTSB, accelerating NLRP3 activation and pyroptosis. Parkin triggers mitophagy and upregulates A20, inhibiting NF-κB and NLRP3. NF-κB, via p62 and Nrf2/ARE, activates Caspase-1, accelerating pyroptosis. AMPK phosphorylation. Degrades TXNIP, activating NLRP3. The AMPK/mTOR pathway upregulates SIRT1, downregulates ROS, and inhibits NLRP3 ↑ induce; ⟂ inhibit.

The interplay between autophagy and pyroptosis in IS is intricate. Autophagy, by degrading damaged cellular components, can foster cell survival and dampen pyroptosis triggers. Nevertheless, exaggerated autophagy may conversely lead to cell demise, which may, in turn, amplify pyroptosis. On the other hand, pyroptosis unleashes inflammatory cytokines, which can ignite autophagy in adjacent cells, fostering a detrimental cycle of cell loss and inflammation (Figure 4).

The interplay between autophagy and pyroptosis in IS. SIRT1 is not only induced by mTOR, it also induces mTOR by inhibiting TSC2 activity, while SIRT3 also activates ATG4B. SIRT1 suppresses ROS in mitochondria, while SIRT3 enhances mitophagy and also reduces ROS levels, thereby inhibiting NLRP3 inflammasome activation. Additionally, NLRP3 expression reciprocally regulates mitochondrial damage and ROS production. Autophagy boosts CTSB, accelerating NLRP3 activation and pyroptosis. Parkin triggers mitophagy and upregulates A20, inhibiting NF-κB and NLRP3. NF-κB, via p62 and Nrf2/ARE, activates Caspase-1, accelerating pyroptosis. AMPK phosphorylation. Degrades TXNIP, activating NLRP3. The AMPK/mTOR pathway upregulates SIRT1, downregulates ROS, and inhibits NLRP3 ↑ induce; ⟂ inhibit.

#### 6.2.1 Mitochondrial reactive oxygen species

The accumulation of mtROS has been intricately linked to the activation of the NLRP3 inflammasome, a component of the NLR family containing the pyrin domain (Liu Z. et al., 2022; Wang et al., 2022). Mitophagy, an autophagic process, specifically targets dysfunctional and potentially harmful mitochondria for degradation. However, when mitophagy is impeded, damaged mitochondria fail to be cleared, leading to the release of mtDNA and mtROS. This, in turn, triggers the activation of the NLRP3 inflammasome. Subsequently, the activated NLRP3 inflammasome can cause mitochondrial membrane rupture, further releasing mtROS and exacerbating mitochondrial damage and inflammation (Dominic et al., 2022). This interaction between mtROS and the NLRP3 inflammasome forms a feedback loop, indicating mtROS’s regulatory function in activating the NLRP3 Inflammasome. Additionally, recent studies indicate the involvement of novel substances in modulating mtROS’s effect on pyroptosis. The study found that Quercetin prevents neuronal injury via inhibition of mtROS-mediated NLRP3 inflammasome activation in microglia through promoting mitophagy (Han et al., 2021).

#### 6.2.2 Parkin, p62, and NF-κB

In recent years, the intricate mechanism underlying the initiation of mitophagy via the PINK1-Parkin pathway has been unveiled (Eckl et al., 2021). Parkin plays a crucial role in suppressing the activation of inflammasomes. Specifically, it enhances the expression of anti-apoptotic signaling protein 20 (A20), subsequently impeding the nuclear entry and activation of nuclear factor kappa-B (NF-κB) (Liu H. et al., 2021; Mouton-Liger et al., 2018). This results in a reduction in NLRP3 inflammasome activity. Additionally, the NF-κB-p62 mitophagy pathway functions to restrain Caspase-1 and mitigate pyroptosis, establishing a reciprocal regulatory mechanism between autophagy and pyroptosis. Recent research has also revealed that Nrf2, a nuclear factor that is activated by p62, is implicated in macrophage pyroptosis. Studies have shown that Glycosides hinder inflammation by inhibiting pyroptosis and p62, while boosting PINK1 and Parkin to activate mitophagy. This results in neuroprotective effects against cerebral ischemia–reperfusion, showing their potential in reducing neurological damage (Jiao et al., 2024).

#### 6.2.3 NLRP3/CTSB

CTSB, a key intracellular cysteine protease primarily localized in lysosomes, exhibits a close link to the autophagic flux within the cytoplasm. Notably, icaritin has been found to alleviate neuroinflammation in brain tissue by intricately modulating the TLR4, MAPK, and NLRP3 inflammasome signaling cascades, with CTSB serving as a pivotal target in this regulatory process (Sun et al., 2023).

#### 6.2.4 Autophagy-related protein

The AMPK/mTOR signaling axis plays a pivotal role in regulating autophagy. Recent studies have demonstrated that eugenol pretreatment mitigates I/R injury by promoting autophagy through the intricate modulation of the AMPK/mTOR/P70S6K signaling pathway (Sun et al., 2020). Moreover, research has elucidated the neuroprotective efficacy of CK against neural autophagy and apoptosis induced by OGD/R, which is achieved through the strategic regulation of the AMPK and mTOR signaling pathways (Huang et al., 2020). Besides, research underscores schaftoside’s neuroprotective capacity against cerebral ischemia/reperfusion injury (CI/RI), both *in vitro* and *in vivo*, by modulating the autophagy-mediated AMPK/mTOR pathway (Zhang L. et al., 2022).

Also, the study has demonstrated that Pien-Tze-Huang inhibited NLRP3 inflammasome-mediated neuroinflammation, which was associated with enhanced autophagy via AMPK/mTOR/ULK1 pathway in vitro and in vivo (Huang et al., 2021).

### 6.3 The interplay of autophagy and necroptosis in IS

**Figure 5:**
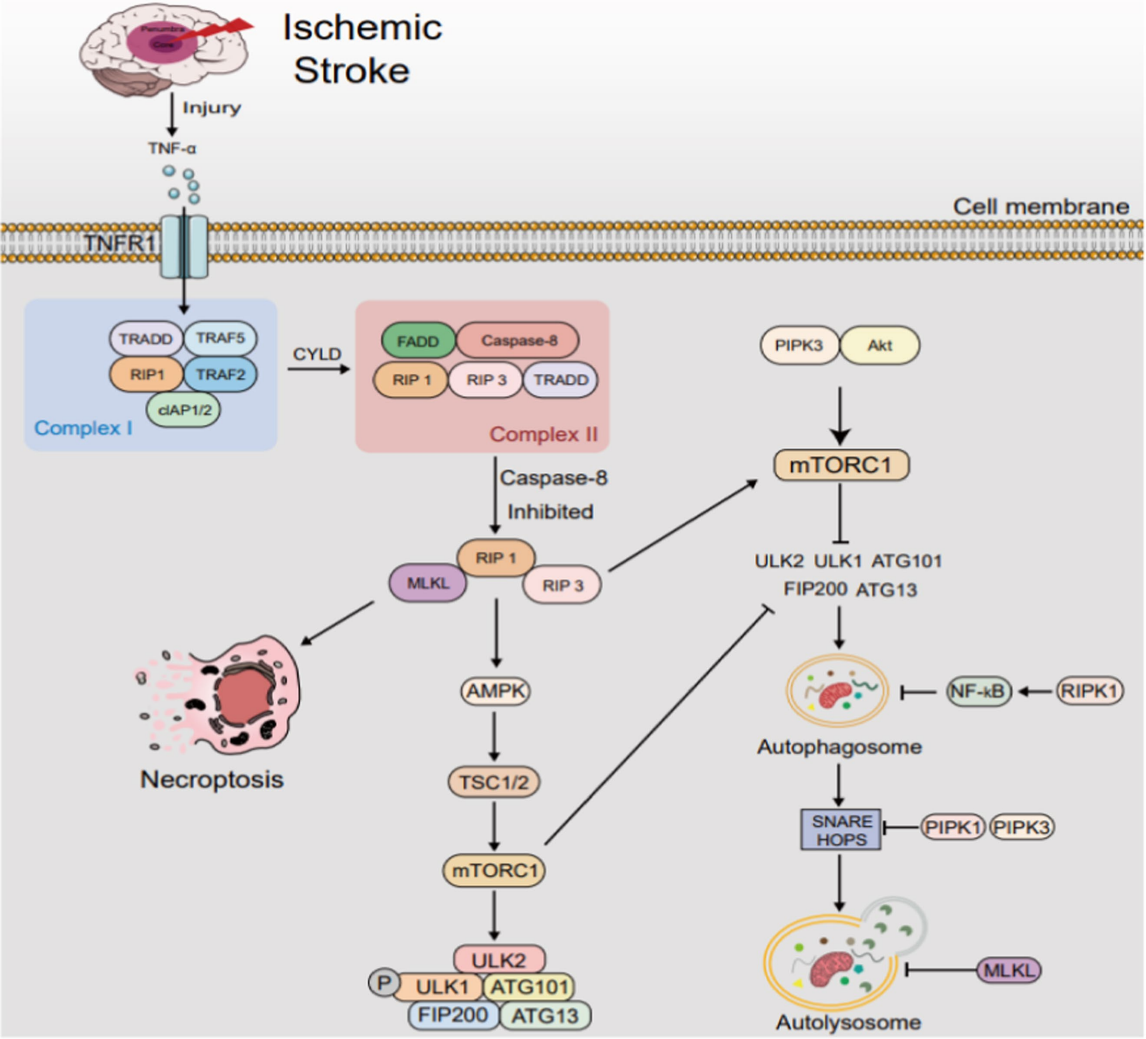
The interplay between autophagy and necroptosis in IS. After IS, TNF-α elevation triggers complex I/II formation, transforming into necrosome under caspase-8 inhibition. This prompts RIP1 activation, RIP3 and MLKL phosphorylation, accelerating necroptosis, which modulates autophagy. RIPK1 and RIPK3 stimulate AMPK, fostering autophagy via the AMPK/TSC/mTORC1 pathway. RIPK3 also phosphorylates ULK1 for alternate autophagy. Conversely, RIPK1 hinders autophagy by activating NF-κB and disrupting autophagosome-lysosome fusion via SNARE complex interference. MLKL inhibits autophagy by activating PI3K/AKT/mTOR signaling and damaging autophagosomal membranes, disrupting autophagic flux. TRADD: TNF receptor-associated death domain; TRAF: TNF receptor-associated factor; FADD: Fas-associating with death domain; cIAP: cellular inhibitors of apoptosis protein. mTOR, mechanistic target of rapamycin; SNARE, soluble N-ethylmaleimide-sensitive factor attachment protein receptor.

The interplay between autophagy and necroptosis in ischemic stroke is characterized by a delicate balance. Autophagy can function as a protective mechanism against necroptosis by eliminating harmful stimuli and promoting cell survival. However, when autophagy is insufficient or overwhelmed, necroptosis may be triggered as a backup mechanism to eliminate severely damaged cells, thereby limiting tissue damage and promoting tissue homeostasis (Figure 5).

The interplay between autophagy and necroptosis in IS. After IS, TNF-α elevation triggers complex I/II formation, transforming into necrosome under caspase-8 inhibition. This prompts RIP1 activation, RIP3 and MLKL phosphorylation, accelerating necroptosis, which modulates autophagy. RIPK1 and RIPK3 stimulate AMPK, fostering autophagy via the AMPK/TSC/mTORC1 pathway. RIPK3 also phosphorylates ULK1 for alternate autophagy. Conversely, RIPK1 hinders autophagy by activating NF-κB and disrupting autophagosome-lysosome fusion via SNARE complex interference. MLKL inhibits autophagy by activating PI3K/AKT/mTOR signaling and damaging autophagosomal membranes, disrupting autophagic flux. TRADD: TNF receptor-associated death domain; TRAF: TNF receptor-associated factor; FADD: Fas-associating with death domain; cIAP: cellular inhibitors of apoptosis protein. mTOR, mechanistic target of rapamycin; SNARE, soluble N-ethylmaleimide-sensitive factor attachment protein receptor.

#### 6.3.1 RIPK1/RIPK3

Recent studies have illuminated the pivotal role that RIPK1 and RIPK3 plays in regulating cellular processes, particularly in the context of amino acid availability, autophagy, and programmed cell death (Tian et al., 2022; Wu X. et al., 2020). RIPK1 and RIPK3 emerge as pivotal players, not merely initiating necroptosis but also intricately intertwining with autophagy signaling pathways to preserve cellular homeostasis. Specifically, they regulate the availability of asparagine, a crucial amino acid that directly influences AMPK (AMP-Activated Protein Kinase) activation—a master switch for autophagy. By modulating asparagine levels, RIPK1 and RIPK3 subtly impact AMPK’s activity, thereby indirectly governing the initiation and progression of autophagy. Autophagy, being a fundamental cellular process, ensures the degradation and recycling of damaged or redundant cellular constituents, thereby maintaining cellular integrity and survival.

Furthermore, RIPK1 and RIPK3 engages in multifaceted interactions with autophagy signaling pathways. It modulates the expression or activity of autophagy-related genes and proteins at multiple levels, including the well-known P13K/AKT/mTOR signaling cascade. Likewise, RIPK1 assumes a pivotal role in cellular adaptation to low energy states, intricately orchestrating the AMPK-mTORC1 signaling cascade (Najafov et al., 2021). This intricate crosstalk between RIPK1 and autophagy signaling underscores the complexity of cellular regulatory networks and highlights the importance of RIPK1 in maintaining these networks’ delicate balance. Beyond its role in autophagy regulation, RIPK1 serves as a critical initiator of necroptosis, a highly regulated form of necrosis that occurs when apoptosis (another form of programmed cell death) is compromised. Upon activation, RIPK1 forms a complex with RIPK3, leading to MLKL phosphorylation and subsequent activation, ultimately triggering necroptosis—a distinct form of cell death marked by membrane disruption and the release of intracellular contents.

Essentially, RIPK1 and RIPK3 function as a gatekeeper, meticulously balancing the intricate equilibrium between inflammation and cell death. Their absence may shift cellular fate towards apoptosis or alternative cell death pathways, potentially jeopardizing tissue integrity and function. Conversely, unchecked RIPK1 activity can spur unchecked inflammation and tissue damage, as necroptosis amplifies inflammatory signals, attracting immune cells to the site of cell demise. Notably, research demonstrates that catalytically inactive RIP1 can mitigate infarct size and enhance neurological recovery following middle cerebral artery occlusion/reperfusion (MCAO/R), highlighting its therapeutic potential in managing the delicate balance between inflammation and cell death (Zhang et al., 2020). Additionally, Oridonin potently halts RIPK3-mediated excessive mitophagy, consequently salvaging neuronal loss at the onset of IS, highlighting its therapeutic potential (Li L. et al., 2023). Furthermore, the study has indicated that RIP3 regulates early reperfusion injury via oxidative stress and mitochondrial activity-related effects, rather than cell loss due to necroptosis (Horvath et al., 2021).

#### 6.3.2 MLKL

Upon its phosphorylation by RIPK3, MLKL undergoes a profound conformational shift. This transformation empowers MLKL to translocate to the plasma membrane, where it disrupts its integrity, ultimately triggering cell death (Fan et al., 2024). This orchestrated sequence underscores the intricate interplay between MLKL’s structural dynamics and its functional role in necroptosis. The activation of MLKL is meticulously controlled by the interplay between RIPK1 and RIPK3, ensuring that necroptosis is initiated only in response to specific stimuli and when apoptotic pathways are impaired. This tight regulation underscores the cell’s meticulous balancing act between life and death decisions. Moreover, necroptosis, orchestrated by RIPK1 and MLKL, has far-reaching consequences beyond mere cell demise. By releasing Damage-Associated Molecular Patterns (DAMPs) and activating immune cells, necroptosis fuels inflammation, highlighting MLKL’s pivotal role in orchestrating the inflammatory cascade associated with this form of cell death.

Intriguingly, while MLKL’s direct involvement in autophagy remains elusive, its function in necroptosis may indirectly influence autophagy processes. By modulating cellular homeostasis and stress responses, necroptosis can potentially alter the cellular landscape in ways that impact autophagy, the cellular ‘recycling’ mechanism. Thus, the story of MLKL extends beyond its primary role in necroptosis, inviting further exploration into its broader implications for cellular physiology and pathology. The investigation reveals that necrosulfonamide safeguards against focal ischemia/reperfusion injury by inhibiting astrocytic necroptosis. This protection occurs through preventing the upregulation of necroptotic kinases and impeding the co-localization of phosphorylated MLKL (p-MLKL) and RIP3K (p-RIP3K) within the nucleus and nuclear envelope. Notably, the translocation of both p-MLKL and p-RIP3K to these compartments is crucial in MLKL-mediated necroptosis under ischemic conditions (Zhou et al., 2023).

## 7 Pharmacotherapies for is targeting autophagy and panoptosis

Currently, the clinical management of IS primarily revolves around interventional approaches to restore blood flow, including drug-based or mechanical thrombolysis techniques. Nevertheless, these strategies have encountered limitations in their effectiveness, and there remains a dearth of efficacious interventions or treatments that can safeguard the brain from cellular demise (Zhou R. P. et al., 2019).

Therapeutic strategies aimed at modulating autophagy present a promising avenue in the management of IS. These strategies encompass both enhancing adaptive autophagy and inhibiting excessive autophagy that occurs post-IS, thereby modulating distinct autophagy processes. Several compounds have demonstrated their ability to induce adaptive autophagy. Notably, rapamycin, extensively studied for its mTOR inhibition properties, has shown potential in managing IS. Recent rodent studies employing middle cerebral artery occlusion (MCAO) models have reported reduced infarct volumes, mitigated neuronal damage, and improved neurological outcomes upon rapamycin administration (Wang S. et al., 2019). Furthermore, resveratrol, a naturally occurring polyphenol, has garnered attention for its ability to extend the therapeutic window of recombinant tissue plasminogen activator (r-tPA) in stroke patients (Chen et al., 2016). Research by He et al. underscores resveratrol’s efficacy in alleviating cerebral ischemia–reperfusion (I/R) injury and reducing infarct size (He et al., 2017). Regarding the inhibition of excessive autophagy, dexmedetomidine (DEX) has emerged as a neuroprotective agent in IS. In both *in vitro* neuronal cultures subjected to oxygen–glucose deprivation (OGD) and *in vivo* transient middle cerebral artery occlusion (tMCAO) models, DEX has demonstrated neuroprotective effects by suppressing autophagy. This protection is mediated through the upregulation of HIF-1α, effectively inhibiting neuronal autophagy and mitigating ischemia–reperfusion injury in the mouse brain (Luo et al., 2017). Propofol administration has been found to significantly reduce infarct size and enhance the prognosis of acute IS patients (Sun B. et al., 2018). Recent research insights indicate that propofol’s neuroprotective effects against CIRI stem from its ability to suppress excessive autophagy, accomplished through the regulation of either the mTOR/S6K1 pathway or the long noncoding RNA SNHG14 (Sun et al., 2021). In parallel, Icariside II, a key bioactive component derived from Epimedii, has shown remarkable neuroprotective potential in OGD and rodent MCAO models. Its mechanism involves curbing excessive autophagy, achieved by modulating the PKG/GSK-3β signaling cascade (Gao et al., 2020). Moreover, Gomisin N (GN) has recently garnered attention for its novel neuroprotective role in mitigating cerebral ischemia injury. This protective effect is postulated to be intimately linked to GN’s ability to inhibit autophagy. Notably, studies suggest that GN’s neuroprotective actions may be mediated through the PI3K/AKT/mTOR signaling pathway, further underlining its therapeutic potential (Li R. Q. et al., 2023).

Therapeutic approaches that modulate pyroptosis, necroptosis, and apoptosis hold great promise in the treatment of IS. Regarding pyroptosis, recent findings have illuminated that Chrysophanol postconditioning protects against cerebral ischemia–reperfusion injury (CIRI) by inhibiting NLRP3-related pyroptosis, a process that is contingent on TRAF6 (Xia et al., 2022). Notably, isoquercetin post-treatment has been shown to mitigate infarct size, reduce the number of apoptotic cells, and alleviate oxidative stress and inflammatory responses following ischemia and reperfusion (Wang J. et al., 2017). Moreover, berberine has been discovered to safeguard rats from cerebral ischemia–reperfusion (I/R) injury by upregulating PPAR-γ, which curbs NF-κB-mediated pyroptosis (Zhao et al., 2021). In a novel finding, we have identified the involvement of pyroptosis in brain microvascular endothelial cells in the pathogenesis of IS. Notably, Medioresinol ameliorates this pyroptosis and reduces ischemic brain injury, potentially through decreasing mitochondrial reactive oxygen species (mtROS) via the PPARα/GOT1 axis in endothelial cells (Wang et al., 2021). Dendrobium has also demonstrated its protective effects against CIR damage by inhibiting pyroptosis-mediated neuronal death (Liu J. et al., 2020). Furthermore, remimazolam has been shown to effectively improve neurological function, reduce infarct volume, and alleviate cortical neuronal damage post-I/R injury. Importantly, the downregulation of the NLRP3 inflammasome pathway suggests that remimazolam exerts its protective effects by suppressing pyroptosis and reducing the expression and release of inflammatory factors (Shi et al., 2022). In the context of necroptosis, the RIP1 inhibitor, necrostatin-1 (Nec-1), effectively decreases RIP1 and RIP3 protein levels, thereby suppressing necroptosis and enhancing cognitive function (Deng et al., 2019; Zhang et al., 2016). Necrosulfonamide, a potent small-molecule inhibitor targeting MLKL, binds to its N-terminal CC region, diminishing MLKL expression and consequently inhibiting necroptosis. This leads to a reduction in infarct volume and improvement in neurological outcomes (Zhou et al., 2017). Dabrafenib, an RIP3 inhibitor at micromolar concentrations, reduces TNF-α mRNA levels and dampens TNF-α activation in macrophages, contributing to a decrease in infarct size and neuronal protection post-focal cerebral ischemic injury (Cruz et al., 2018). Infliximab, a monoclonal antibody commonly utilized in inflammatory diseases, has been shown to mitigate mitochondrial damage, limit cytoplasmic transparency, reduce blood–brain barrier (BBB) permeability, and inhibit necroptosis formation in ischemic regions, ultimately ameliorating neurological deficits (Chen et al., 2019). Furthermore, the inhibition of necroptosis-related gene expression is being actively explored as a therapeutic strategy for IS. For instance, the combination of Gsk′872 (an RIP3 inhibitor) and RIP3 siRNA reduces the levels of RIP1, RIP3, MLKL, and phosphorylated MLKL, offering neuroprotective benefits. Chinese herbs and their active ingredients have also garnered attention for their ability to decrease necroptosis-related proteins (RIP3, MLKL, and phosphorylated MLKL), suppressing necroptosis and enhancing neurological function in rats subjected to middle cerebral artery occlusion (MCAO) (Zhang D. M. et al., 2019). Notably, Panax notoginseng saponins, isolated from the plant’s roots, inhibit necroptosis by downregulating the expression of these necroptosis-associated proteins (Hu et al., 2022). In the realm of apoptosis, studies have unveiled that Salidroside exerts a protective effect against CIRI-induced microglial NLRP3 inflammasome activation and apoptosis by inhibiting the TLR4/NF-κB signaling cascade (Liu et al., 2021). Meanwhile, Astilbin safeguards against CIRI by modulating the MAPK pathway (suppression) and AKT pathway (activation), thereby inhibiting apoptosis and inflammation (Li et al., 2020). Furthermore, myricetin has been shown to significantly reduce neuronal apoptosis both *in vivo* and *in vitro* following ischemic events. Notably, myricetin alleviates various apoptotic factors associated with IS, including excitotoxicity, oxidative stress, and inflammation-mediated apoptosis (Zhang L. et al., 2023).

Additional pharmacotherapeutics aimed at modulating pyroptosis, apoptosis, necroptosis and autophagy in IS have garnered substantial research attention. Specifically, regarding autophagy and pyroptosis, Astragaloside IV demonstrates efficacy in reducing cerebral infarct volume and neurological deficit scores, while enhancing cell viability and regulating autophagy, with an inhibitory effect on pyroptosis (Xiao et al., 2021; Zhang Y. Y. et al., 2019). Spautin-1, on the other hand, is postulated to alleviate cerebral ischemia–reperfusion injury via modulation of the autophagy/pyroptosis pathway (Liu et al., 2021a,b). When it comes to autophagy and necroptosis, URB597 exhibits a dose-dependent improvement in neurological function and reduction in brain infarct volume and edema post-ischemia. It also enhances autophagic flux and mitigates neuronal necroptosis (Yuan et al., 2023). Cyclosporine-A, another compound, effectively diminishes autophagy-related proteins while reducing necroptosis markers like RIP1 and RIP3 (Fakharnia et al., 2017). Baf-A1 treatment is found to mitigate necroptosis and block autophagy, while global cerebral ischemia/reperfusion (I/R) induces necroptosis, possibly augmented by autophagy, and inversely influences caspase-3-mediated apoptosis (Ryan et al., 2018). Regarding autophagy and apoptosis, melatonin administration post-cerebral ischemia (CI) reduces infarct area and induces autophagic proteins like Beclin-1, LC3, and p62 by inhibiting the apoptotic caspase-3 protein (Yilmaz et al., 2023). Resveratrol pretreatment, too, extends the therapeutic window in cerebral ischemic mice, modulating autophagy and inhibiting apoptosis (He et al., 2017; Hou et al., 2018). 6-Gingerol exerts its anti-apoptotic and anti-inflammatory effects via TRPV1/FAF1 complex dissociation-mediated autophagy during cerebral I/R injury (Luo et al., 2021). Furthermore, Stilbene glycoside promotes mitochondrial autophagy in ischemic neurons by upregulating SIRT3/AMPK expression, thereby inhibiting apoptosis (Li et al., 2021). Notably, the study has demonstrated neuroprotective potential of safinamide via anti-oxidant, anti-inflammatory, anti-apoptotic, and autophagy inducing properties (Wasan et al., 2021). Table 2 presents data pertaining to pharmacotherapeutic targets aimed at modulating PANoptosis and autophagy in the context of cerebral ischemia or IS, offering insights into potential therapeutic strategies.

**Table 2:** Pharmacotherapies target in PANoptosis and autophagy against cerebral ischemia or IS.

Pharmacotherapy	Subject	Effects	References
Autophagy			
Rapamycin	MCAO rats and mice	Reduces endothelial cell death, neuronal damage, infarct size and enhance neurological recovery.	Wang J. F. et al. (2019)
Resveratrol	tMCAO rats	Inhibits neuronal autophagy and enhances neurological recovery.	He et al. (2017)
Propofol	MCAO rats	Reduces the infarct volume, inhibits excessive autophagy through regulation of mTOR/S6K1 or long noncoding RNA SNHG14.	Sun et al. (2021)
Icariside II	MCAO rats; OGD-subjected, primary cortical neurons	Protects neurons, inhibits excessive autophagy via interfering with the PKG/GSK-3β signaling pathway.	Gao et al. (2020)
Gomisin N	MCAO/R mice; OGD/R-subjected	Protects neurons, inhibits autophagy through via with the PI3K/AKT/mTOR signaling pathway.	Li R. Q. et al. (2023)
Pyroptosis			
Chrysophanol	MCAO mice	Protective against CIRI by inhibiting NLRP3-related pyroptosis in a TRAF6-dependent manner.	Xia et al. (2022)
Isoquercetin	MCAO/R rats; OGD/R-subjected	Reduces the infarct volume, number of apoptotic cells.	Wang C. P. et al. (2017)
Berberine	OGD	Protects rats from cerebral I/R injury, reduces pyroptosis.	Zhao et al. (2021)
Medioresinol	tMCAO mice; OGD/R	Inhibits pyroptosis.	Wang et al. (2021)
Dendrobium alkaloids	OGD/R	Inhibits pyroptosis, reduces the infarct size.	Liu J. et al. (2020)
Remimazolam	MCAO/R rats	Improves the neurological dysfunction, and alleviate the damage of cortical neurons after I/R, reduces the infarct size injury, suppresses pyroptosis.	Shi et al. (2022)
Necroptosis			
Nec-1	BCAS mice	Inhibits RIP1 and RIP3 to reduce inflammation and enhances cognitive function.	Zhang et al. (2016)
Nec-1	MCAO rats	Decreases phosphorylated RIP1, RIP3, MLKL, and phosphorylated MLKL levels and the numbers of phosphorylated RIP1^+^ neurons.	Deng et al. (2019)
Necrosulfonamide	tMCAO mice	Reduces MLKL expression and infarct size and enhances neurological function.	Zhou et al. (2017)
Dabrafenib	Focal ischemic brain injury model mice	Reduces TNF-α mRNA levels and infarct size.	Cruz et al. (2018)
Infliximab	MCAO rats	Reduces mitochondrial damage, cytoplasm transparency, and BBB permeability.	Chen et al. (2019)
Gsk′872 + RIP3 siRNA	MCAO mice; OGD-subjected HT-22 cells	Reduces RIP1, RIP3, MLKL, and phosphorylated MLKL levels to protect the neurological system.	Yang et al. (2017)
Ligustroflavone	MCAO rats	Reduces RIP3, MLKL, and phosphorylated MLKL levels to enhance neurological function.	Zhang Y. Y. et al. (2019)
Panax notoginseng saponins	MCAO rats	Decreases the expression of necroptosis-associated proteins.	Hu et al. (2022)
Apoptosis			
Salidroside	MCAO/R; OGD/R	Reduces cerebral infarction size, and inhibits apoptosis.	Liu et al. (2021)
Astilbin	MCAO; OGD	Against cerebral I/R injury by inhibiting apoptosis and inflammation via suppressing the MAPK pathway and triggering the AKT pathway., reduces apoptosis.	Li et al. (2020)
Myricetin	MCAO; OGD	Reduces neuronal apoptosis after ischemia *in vivo* and *in vitro*.	Zhang H. et al. (2023)
Autophagy and Pyroptosis			
Astragaloside IV	MCAO/R rats; OGD/R-subjected	Reduces the cerebral infarct size and the neurological deficit score in vivo and increases the cell viability, inhibition of pyroptosis and regulation of autophagy.	Xiao et al. (2021); Zhang Y. et al. (2019)
Spautin-1	MCAO/R rats; OGD/R	Reduces autophagy and ROS accumulation and attenuates NLRP3 inflammasome-dependent pyroptosis	Liu et al. (2021)
Autophagy and necroptosis			
URB597	MCAO mice	Improves neurological function and reduces brain infarct size, attenuates autophagic flux and reduces neuronal necroptosis	Yuan et al. (2023)
Cyclosporine-A	The 4-vessel occlusion rats	Decreases autophagy associated proteins, reduces in necroptosis markers, RIP1 and RIP3	Fakharnia et al. (2017)
Bafilomycin-A1	The 4-vessel occlusion rats	Attenuates necroptosis, inhibits autophagy	Ryan et al. (2018)
Autophagy and apoptosis			
Melatonin	MCAO mice	Reduces the infarct area and induces the autophagic proteins Beclin-1, LC3, and p62 via inhibiting the apoptotic caspase-3 protein.	Yilmaz et al., (2023)
Resveratrol	MCRO/R; OGD	Regulation of autophagy, and anti-apoptosis	He et al. (2017); Hou et al. (2018)
6-Gingerol	MCAO/R; OGD/R	Inhibits apoptosis and upregulates autophagy	Luo et al. (2021)
Stilbene glycoside	OGD	Promotes mitochondrial autophagy in ischemic neurons and inhibits apoptosis.	Li et al. (2021)
Safinamide	MCAO/R rats	Reduces the cerebral infarct size, anti-oxidant, anti-inflammatory, anti-apoptotic, and autophagy	Wasan et al. (2021)

BCAS, bilateral common carotid artery stenosis.

## 8 Conclusion and perspectives

Stroke is assuming a pivotal role as a major concern for individuals in developing nations, particularly in China, where IS stands as the foremost cause of mortality due to its staggeringly high morbidity, mortality, and disability rates. Multiple cell death pathways have been identified, and extensive research has illuminated their significance in maintaining biological homeostasis, as well as the intricate interplay between them. Elucidating the molecular mechanisms underlying this crosstalk will not only enhance our comprehension of the cell death machinery but also offer potential novel therapeutic targets for related illnesses. The intricate crosstalk between PANoptosis and autophagy in the context of IS has garnered significant attention in recent years, owing to its profound implications in neuronal survival and recovery. PANoptosis, as a novel form of cell death, encompasses necroptosis, apoptosis, and pyroptosis, each with unique mechanisms and downstream effects. Autophagy, on the other hand, serves as a crucial cellular mechanism for the degradation and recycling of damaged components, playing a pivotal role in maintaining cellular homeostasis.

The current understanding of the interplay between these two processes in IS suggests that they are intricately linked and mutually influential. On one hand, PANoptosis can be triggered by ischemia-induced stress, leading to neuronal cell death and tissue damage. On the other hand, autophagy can either promote cell survival by clearing damaged proteins and organelles or contribute to cell death if.

excessive or deregulated. The identification of novel therapeutic targets that can modulate this crosstalk holds immense promise for the development of effective treatments for IS. For instance, targeting specific PANoptosis pathways or enhancing autophagic flux may offer neuroprotective effects, reducing infarct size and improving functional recovery. However, the complex nature of these pathways necessitates a cautious and thorough approach, as modulation of one process may inadvertently affect the other. Future research should focus on elucidating the precise molecular mechanisms underlying the crosstalk between PANoptosis and autophagy in IS. This will not only provide a deeper understanding of the disease but also aid in the identification of novel therapeutic targets. Furthermore, preclinical and clinical studies are essential to validate the efficacy and safety of potential therapies.

In conclusion, the intersection between PANoptosis and autophagy represents a fertile ground for the development of novel therapeutic strategies in IS. With continued research and innovation, we may soon witness a paradigm shift in the treatment of this debilitating disease.
